# Magnesium diboride coated bulk niobium: a new approach to higher acceleration gradient

**DOI:** 10.1038/srep35879

**Published:** 2016-10-24

**Authors:** Teng Tan, M. A. Wolak, X. X. Xi, T. Tajima, L. Civale

**Affiliations:** 1 Department of Physics, Temple University, Philadelphia, 19122, Pennsylvania, USA; 2 Los Alamos National Laboratory, Los Alamos, 87545, New Mexico, USA.

## Abstract

Bulk niobium Superconducting Radio-Frequency cavities are a leading accelerator technology. Their performance is limited by the cavity loss and maximum acceleration gradient, which are negatively affected by vortex penetration into the superconductor when the peak magnetic field at the cavity wall surface exceeds the vortex penetration field (*H*_vp_). It has been proposed that coating the inner wall of an SRF cavity with superconducting thin films increases *H*_vp_. In this work, we utilized Nb ellipsoid to simulate an inverse SRF cavity and investigate the effect of coating it with magnesium diboride layer on the vortex penetration field. A significant enhancement of *H*_vp_ was observed. At 2.8 K, *H*_vp_ increased from 2100 Oe for an uncoated Nb ellipsoid to 2700 Oe for a Nb ellipsoid coated with ~200 nm thick MgB_2_ thin film. This finding creates a new route towards achieving higher acceleration gradient in SRF cavity accelerator beyond the theoretical limit of bulk Nb.

Particle accelerators are our main window to explore particle physics and understand the early universe. Bulk niobium superconducting radio frequency (SRF) cavities are the leading accelerator technology, and in the last 2 decades, their performance has been improved to being very close to the theoretical limit. New materials and/or approaches are needed to further improve the performance and reduce the cost of SRF cavities^1^,^2^. In this work, we studied the magnesium diboride (MgB_2_) coated bulk niobium and showed the coated SRF cavities have the potential to reach 30% higher acceleration gradient than the state of the art Nb cavities.

The technology of SRF cavities is based on the Meissner effect, characterized by the expulsion of magnetic field from a bulk superconducting material. In type II superconductors, such as Nb, the Meissner state can only be sustained up to a certain magnetic field, above which flux will penetrate the material in the form of vortices. In the presence of an RF excitation these vortices will oscillate, producing dissipation that drastically reduces the quality factor (*Q*), thus rendering the cavity useless. The limiting factor is the maximum accelerating gradient (*E*_acc_), which is related to the maximum magnetic field parallel to the cavity surface that can be sustained without vortex penetration (*H*_vp_). In thermodynamic equilibrium, *H*_vp_ of a bulk superconductor in the absence of demagnetizing effects is equal to the lower critical field (*H*_c1_). However, surface barriers may preclude the vortex entry, allowing the Meissner state be retained under metastable conditions up to a higher superheating field *H*_sh_^3^. The best Nb cavities have *E*_acc_ 50 MV/m at temperature *T*~2 K (the operating temperature of the SRF cavities at *f* > 500 MHz), corresponding to *H*_vp,Nb_~2000 Oe^4^, which is very close to the ideal limit set by the intrinsic material properties, *H*_sh,Nb_(2 K) ≈ 2280 Oe^5^.

To increase *H*_vp_, Gurevich proposed coating the inside of Nb cavities with multilayers of thin superconducting films separated by insulating layers^2^. The idea is that the superconducting films will partially screen the cavity magnetic field, reducing it to a value lower than *H*_vp,Nb_ at the internal surface of the Nb wall. We will focus on the simplest case of a SRF cavity coated with a single layer of superconductor, because this S-I-S structure already exhibits all the relevant physics and detailed theoretical analyses of its shielding effect have recently been performed with various approaches^6^,^7^,^8^,^9^.

The principle of operation of the S-I-S structure involves two concepts. First, when the thickness of the superconducting film (*d*_S_) is comparable to or smaller than the penetration depth (λ), *H*_c1_ for field parallel to the surface increases. This is because the internal field is nonzero everywhere within the thin film, thus the free energy cost of shielding the external field by a screening current is lowered and vortex penetration does not become favorable until a higher field. For instance, if *H* is applied on both sides of a film with *d*_S_ ≪ λ, *H*_c1_ increases from the bulk values *H*_c1_ ∝ Φ_0_/λ^2^ to *H*_c1_ ∝ Φ_0_/*d*_S_^2^ (here Φ_0_ = 2.07 × 10^−7^ Gcm^2^ is the flux quantum). The situation is somewhat different when the field is only applied on one side^6^, but also in this case *H*_c1_ for a thin film is substantially enhanced, resulting in a higher *H*_vp_ for the film-coated Nb cavity^10^. The second concept is that the screening (reduction) of the field produced by the film is Δ*H*~*d*_S_*J*, where the superconducting current density (*J*) depends on the details of the geometry but cannot exceed the superconducting depairing current density (*J*_d_) in the film, *J*_d_~*H*_c_/λ, where *H*_c_ is the thermodynamic critical field (*H*_c_ > *H*_c1_ in type II superconductors). It is clear from the previous considerations that if *d*_S_ is too large there is no enough *H*_c1_ enhancement and if it is too small *H*_vp_ is only increased by a small amount Δ*H*, so there must be some optimum *d*_S_ that produces the largest *H*_vp_. T. Kubo^9^,^11^ has shown that, when the simple estimate of an exponential decay of the magnetic field from the film surface is replaced by the simultaneous solution of the Maxwell and London equations in the complete S-I-S structure with the appropriate boundary conditions, *H*_vp_ depends on the properties of both the coating layer and bulk superconducting materials, on *d*_S_, and on the thickness of the insulating layer (*d*_I_). In particular, these studies show that the shielding effect still exists for *d*_I_ = 0, that is, without an insulating layer between the thin film and the niobium underneath. In this paper we provide the first experimental demonstration of the concept, by showing that a Nb SRF cavity coated with a single MgB_2_ layer could sustain a higher *H*_vp_, above the theoretical limit given by the *H*_sh_ for Nb. This could lead to SRF cavities with higher accelerating gradients.

The increase of *H*_c1_ in thin films for **H**//surface is a well-established and well understood effect^10^, and can be readily observed using a SQUID magnetometer. As *H* is increased from 0 at a fixed temperature, the initial Meissner state is characterized by a linear *m(H*) dependence *m*_M_ = −(*V*_eff_/4π)*H*. Here *V*_eff_ is the effective superconductor volume, which is smaller than the geometrical volume *V* due to the field penetration near the surfaces. At *H*_c1_, *m(H*) deviates from the linear dependence, signaling vortex penetration. The *H*_c1_ enhancement in thin MgB_2_ films has been studied previously using this technique^12^. *V*_eff_ for δ ≪ λ is very small, thus the experimental resolution of the magnetometers is a serious limiting factor for these measurements.

However the second concept of Gurevich proposal, namely that a thin film can sustain a Δ*H* between both of its surfaces, cannot be tested using the approach described above because both surfaces of the film are exposed to the same ambient field. In this paper we propose a novel approach that overcomes this fundamental limitation, and concomitantly also solves the resolution and alignment problems (see supplementary information). We have fabricated macroscopic-sized ellipsoids and coated them with MgB_2_ films. The Meissner effect in the film screens the entire ellipsoid volume from the applied field, so a Δ*H* develops between the internal and external film surfaces. Here the field is outside of the ellipsoid rather than inside as in a real SRF cavity, thus we call it the “inverse cavity” concept. We use ellipsoids because the demagnetizing effects and boundary conditions are well defined, and in the Meissner state the external field at the ellipsoid surface is parallel to the surface everywhere, the same as in real SRF cavities.

The Nb ellipsoids have the nominal semi axes *a* = 4 mm, *b* = *c* = 2.5 mm, made from the same bulk material as presently used in SRF cavities. The measurements were performed in a Quantum Design MPMS SQUID magnetometer. When the applied field ***H*** is parallel to the *i* axis (*i* = *a, b, c*) the calculated “Meissner slope” is *m*_M,*i*_/*H* = −*V*/[4π(1 − *N*_*i*_)] = −*ab*^2^/[3(1 − *N*_*i*_)], where *N*_*i*_ are the demagnetizing factors, such that *N*_*a*_ < *N*_*b*_ = *N*_*c*_ and *N*_*a*_ + *N*_*b*_ + *N*_*c*_ = 1.

Figure 1a shows the schematic of the *H*_vp_ measurement. Most results in this paper were measured with the applied magnetic field parallel to the long axis of the ellipsoid (top). However, the field alignment to the short axis (bottom) was also measured to confirm the results taking into account the different demagnetizing factors for the two field directions. Figure 1b presents *m* vs. *H* at several temperatures for a bare Nb ellipsoid shown in the inset. For each *T*, the sample was cooled from above *T*_c_ = 9.3 K to *T* in *H* = 0 (zero field cooling, ZFC) before the *m* vs. *H* measurement. The Meissner slope does not change with *T* because the variation of the effective superconductor volume due to the change in λ(*T*) is too small compared to the dimensions of the ellipsoid. The solid line in this figure corresponds to the predicted *m-H* relationship for the ellipsoid with *a* = 4 mm and *b = c = *2.5 mm in the Meissner state. The *m* vs. *H* curves at *T* = 5 K for the Nb ellipsoid are shown in the inset of Fig. 1c for both **H**||*a* and **H**⊥*a*. From the slopes of both orientations *m*_M,*a*_/*H* and *m*_M,*b*_/*H*, we obtained *N*_*a*_ = 0.1880 and *N*_*b*_ = *N*_*c*_ = 0.4060. The ellipsoid volume was calculated to be *V* = 0.1062 cm^3^, in excellent agreement with the designed value (from the weight of this particular ellipsoid we had determined *V* = 0.1048 cm^3^).

Although the applied field is uniform, due to the demagnetizing effects the total external field is not. In the Meissner state the total external field at the surface of the ellipsoid is parallel to the surface everywhere, and its magnitude varies along the surface, being zero at the poles and reaching a maximum at equator *H*_eq_ = *H*/(1 − *N*_*i*_), where *i* = *a, b*. (see Fig. 1a). Consequently, vortex penetration starts at the equator. As seen in the Fig. 1c, the average magnetic induction <*B*_*i*_> = 4π(1 − *N*_*i*_)(*m* − *m*_M_)/*V* of the ellipsoid starts to deviate from zero at the same *H*_eq_ for both **H**||*a* and **H**||*b* orientations. We define the vortex penetration field *H*_vp_ as when <*B*> increases above the noise. The noise level increases with *H*, reaching about ±2 G at 1.5 kG, which negatively impact the accuracy of the *H*_vp_(*T*) measurement^13^. This high noise level could be originated from the superconducting magnet in the magnetometer when it was charged between 1000 and 5000 Oe with fine steps^14^.

An alternative approach to measure *H*_vp_ is to apply a field *H* < *H*_vp_(*T*) after ZFC and then measure *m(T*). A result is shown in Fig. 1d. As *T* increases, *m* initially remains constant at the Meissner value until the vortex penetration occurs, which is signaled by a sudden reduction of the absolute value of *m* when *H* = *H*_vp_(*T*). Figure 1e summarizes the *H*_vp_(*T*) results obtained from both *m(H*) and *m(T*) measurements. The left and right vertical axes in Fig. 1e show the value of *H* and *H*_eq_ for first vortex penetration respectively. The error bars in Fig. 1e stand for the *H*_vp_ variation when using 5 G and 1 G <*B*> criterion. The results suggest that the *m-T* approach is a preferred method for the *H*_vp_ measurement. At high field, it is more accurate than the *m-H* technique mainly due to the elimination of the flux creep effect from the superconducting magnet. At low *H* it is comparable to the *m-H* technique. Therefore, the *m-T* technique was used to measure *H*_vp_ of a bare Nb ellipsoid and two MgB_2_ thin film-coated Nb ellipsoids in this work. From Fig. 1e we find that at *T*~2 K vortex penetration starts at *H*_eq_~2200 Oe, very close to the ideal limit *H*_sh,Nb_(2 K) ≈ 2280 Oe, confirming the high quality of the Nb used in our study.

The MgB_2_ thin film coating of Nb ellipsoids was performed using hybrid physical-chemical vapor deposition (HPCVD)^15^. Two Nb ellipsoids were coated with MgB_2_ layers of 100 nm (Nb100) and 200 nm (Nb200), respectively. Figure 2a shows an SEM image taken at the equator of the ellipsoid Nb100. The film is polycrystalline with grain size of ~1 μm. The MgB_2_ layer covered the entire ellipsoid uniformly without pinholes. An optical image of Nb200 is presented as the inset to Fig. 2b. Figure 2c shows the *m-T* curves for the bare Nb ellipsoid and the Nb ellipsoids coated with 100 nm and 200 nm MgB_2_ layers for 1500 Oe applied field. The onset of vortex penetration are marked with black arrows in the figure. With an increasing temperature, vortices penetrate into the bare Nb ellipsoid at 3.76 K. This temperature increases to 5.11 K and 5.21 K for the Nb100 and Nb200 ellipsoid, respectively. By repeating the *m-T* measurements, the temperature dependence of *H*_vp_ can be obtained.

Figure 2d summarizes the *H*_vp_(*T*) curves for the bare Nb ellipsoid, Nb100, and Nb200. The three data points within the blue box correspond to the 3 curves in Fig. 2c. The *H*_vp_(*T*) of Nb200 is about 400 Oe higher than the bare Nb ellipsoid in the temperature range from 1.8 K to the *T*_c_ of Nb. This increase is approximately constant because the properties of MgB_2_ do not change significantly in this temperature range (*T* < *T*_c_/4). The maximum field at the equator that our inverse cavity can sustain before vortex penetration is indicated on the right vertical axis. At *T*~2.8 K it is ~2600 Oe, about 500 Oe above the bare Nb ellipsoid.

As an additional test of our inverse cavity concept we deposited MgB_2_ films of thickness *d*_S_ = 100; 200 and 300 nm on non-superconducting molybdenum ellipsoids. For details of the measurements and analysis, see the supplementary material. For the three samples we measured *m(H*) at several *T* with **H**||*a*. The Meissner slopes indicate that the full volume of the ellipsoids is screened, implying that the field at the MgB_2_-Mo interface is zero. At *H*_vp_(*T*) the field at the interface becomes nonzero and spreads inside the Mo. The obtained *H*_vp_(*T*) curves are shown in Fig. 3a. They extrapolate to zero at the *T*_c_ of the MgB_2_ films (~38.1; 38.6 and 38.7 K respectively).

As described in detail in the Supplementary Materials, we found out that in all cases *H*_vp_ at the equator is lower than the prediction for the enhanced *H*_c1_, and that the field penetration occurs when the screening current is large enough to eliminate the surface barrier. The fact that *H*_vp_ in the MgB_2_ coated Mo ellipsoids is determined by *J* rather than *H*_c1_ is good news for cavity applications, because it implies that *H*_vp_ is the limit for the *difference* in field that can be sustained between both surfaces of the film, not for the *absolute value* of the field. This is fully consistent with our main experimental finding, namely that by adding an MgB_2_ coating we have been able to increase *H*_vp_ by about 500 Oe on top of the already large penetration field of Nb.

We performed additional studies on the Mo200 ellipsoid to estimate how high *H*_vp_ could potentially be obtained. After applying a field *H*_int_ at a temperature above the *T*_c_ of MgB_2_ (which penetrates homogeneously into the Mo), we cooled down to a measurement *T* and subsequently increased *H*. We observed an initial linear *m(H*) Meissner dependence, indicating that screening currents develop in the MgB_2_ that shield the internal Mo volume from the external field variation. Eventually the surface barrier disappears and the field penetrates when the difference between the outside and the inside field, Δ*H* = *H* − *H*_int_, reaches a value Δ*H*_vp_, as summarized in Fig. 3b. The remarkable result is that Δ*H*_vp_ > 0 even for *H*_int_ well above the maximum operating fields of Nb cavities. This implies that the operating field of a cavity could be increased by Δ*H*_vp_/(1 − *N*_*a*_) by internally coating it with this MgB_2_ film. For instance, Fig. 3b shows that if a hypothetical cavity were able to sustain *H*_vp_~4000 Oe, at 1.8 K, then this MgB_2_ film would further increase that *H*_vp_ by ~200 Oe.

Our results demonstrate three important facts. First, it is indeed possible to increase the *H*_vp_ of the Nb material used in the state-of-the-art SRF cavities by a substantial amount (~600 Oe) by coating it with a thin superconducting film that screens vortex penetration. Second, it is technically possible to deposit a thin MgB_2_ film with appropriate properties for this purpose on curved bulk Nb. Third, a single layer of superconducting thin film can reduce the magnetic field felt by the bulk material under it. These three facts are enough to demonstrate that it is possible to increase the *H*_vp_ of existing Nb SRF cavities by ~600 Oe. The superconducting shielding current density at the equator of Nb200 is estimated to be *J*~Δ*H*/*d*~10^7^ A/cm^2^, as compared to *J*_d_~10^8^ A/cm^2^ reported for MgB_2_ thin films^16^, therefore the maximum Δ*H* that a MgB_2_ thin film can shield has not been reached. Further, a superconductor/insulating multilayer coating proposed by Gurevich would achieve even higher shielding field Δ*H*_total_ with MgB_2_ films.

## Methods

### Deposition of MgB_2_ thin film on Nb ellipsoids

The MgB_2_ films were deposited by Hybrid Physical-Chemical Vapor Deposition (HPCVD) technique^15^. The schematic of the deposition technique is presented in Extended Data Fig. 1(a). By heating magnesium pellets above melting temperature and letting magnesium vapor react with boron from the decomposition of diborane gas, this technique has produced the films with the highest quality on both single crystal and metallic substrates^17^,^18^. In this work, MgB_2_ film was deposited at 730 °C in 40 Torr hydrogen atmosphere. The boron source was 20 sccm 5% diborane in hydrogen solution, which gave a growth rate of 55 nm/min. Each ellipsoid was coated three times with rotating 120 degree between each coating to achieve uniform coating. During the first deposition, a piece of 5 × 5 mm^2^ silicon carbide (SiC) substrate is put aside of the ellipsoid as a reference for thickness calibration. The nominal thickness of MgB_2_ layer coated on the ellipsoid refers to the film thickness on the SiC substrate.

## Additional Information

**How to cite this article**: Tan, T. *et al*. Magnesium diboride coated bulk niobium: a new approach to higher acceleration gradient. *Sci. Rep.* **6**, 35879; doi: 10.1038/srep35879 (2016).

## Supplementary Material

Supplementary Information

## Figures and Tables

**Figure 1:**
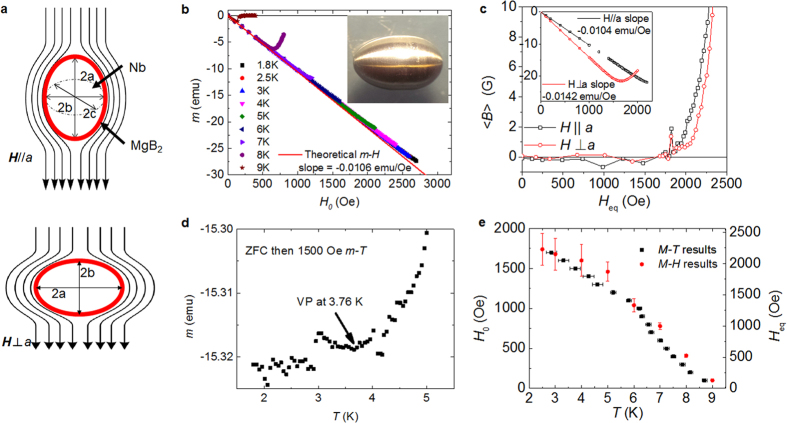
(**a**) Schematic of the vortex penetration field measurement. (**b**) ZFC *m-H* curves of bare Nb ellipsoid at different temperatures. (**c**) <*B*>-*H* curves of bare Nb ellipsoid at different orientations. (**d**) Typical ZFC *m-T* curve of bare Nb ellipsoid under 1000 Oe applied field. Arrow signals the temperature where *H*_vp_ equals the applied field. (**e**) Comparison of *H*_vp_ obtained from both methods.

**Figure 2:**
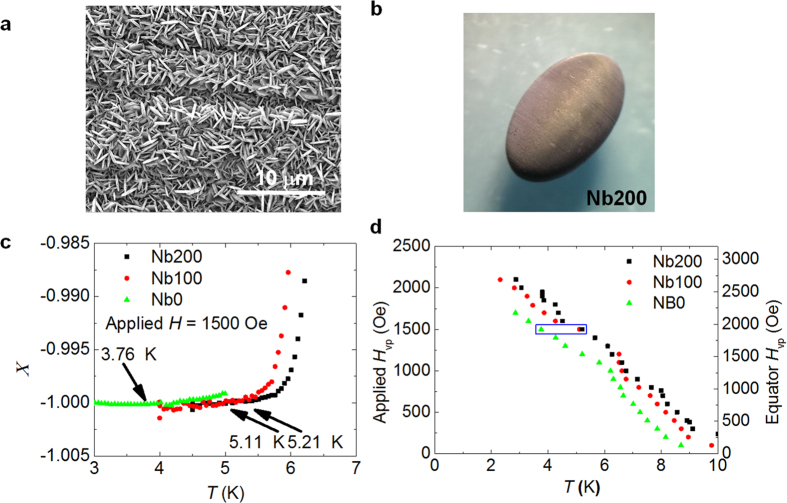
(**a**) SEM image taken on the Nb100 ellipsoid. (**b**) optical image of Nb200 ellipsoid (**c**) *m-T* curves comparisons between Nb ellipsoids with different thicknesses of MgB_2_ coating. ZFC to 1.8 K then apply 1500 Oe field. Arrows indicate the *H*_vp_ of each sample. (**d**) Comparison of *H*_vp_-*T* curves measured on Nb ellipsoids. Data points in the blue box corresponds to the 3 curves in Fig. 2(c). The actual *H* field at the ellipsoids equator is calculated on the right *y*-axis.

**Figure 3:**
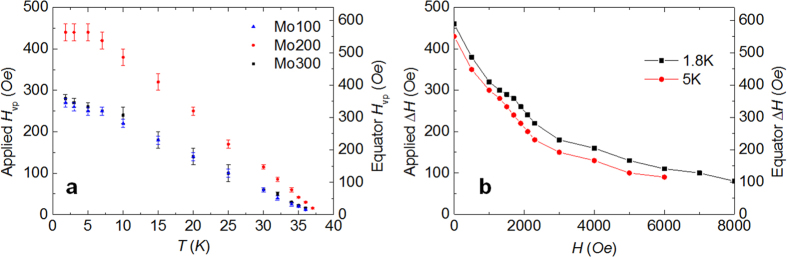
(**a**) The dependence of *H*_vp_ of the MgB_2_/Mo samples on temperature. *H*_vp_s of Mo100 and Mo300 are almost overlapped. (**b**) Maximum *H* difference shieled by a single layer of MgB_2_ versus the field inside the MgB_2_ shell for Mo200 at 1.8 K and 5 K.
