# Supplementary material for: Magnesium diboride coated bulk niobium: a new approach to higher acceleration gradient
Source: Sci Rep. 2016 Oct 24;6:35879. doi: 10.1038/srep35879 (PMC5075871; doi:10.1038/srep35879)
Supplement: Supplementary Information [file srep35879-s1.pdf]

Magnesium diboride coated bulk niobium: a new approach to higher acceleration gradient

Teng Tan[1], M. A. Wolak[1], X. X. Xi[1], T. Tajima[2] and L. Civale*[2]

1. Department of Physics, Temple University, Philadelphia, Pennsylvania, 19122

2. Los Alamos National Laboratory, Los Alamos, New Mexico, 87545

* e-mail: lcivale@lanl.gov

Supplementary Information:

Supplementary Discussions

Resolution limitations and misalignment effects in thin films and ellipsoids

For a square film of side L and thickness δ, in the Meissner state in a parallel field $m_M = -(V_{eff}/4\pi)H$ with $V_{eff} = L^2\delta_{eff} = L^2\delta[1-(2\lambda/\delta)tanh(\delta/2\lambda)]$. For $L \sim 0.5$cm and $\delta_{eff} \sim 100$nm (e.g., $\delta \sim 200$nm and $\lambda \sim 50$nm) results $dm_M/dH \sim 2\times10^{-7}$ emu/Oe, which is around the practical resolution limit of commercial SQUIDs. In the thin film coated ellipsoids, when the film is in the Meissner state the entire ellipsoid volume is screened from the applied field, thus for $L \sim b$ the signal is increased by a factor of the order b/δ, which amounts to several orders of magnitude, opening up the possibility to investigate very thin films.

In thin film measurements the alignment between the film and **H** is critical. A misalignment between **H** and the surface by an angle θ produces a field component normal to the film $H_\perp \sim H\theta$ that due to the huge aspect ratio $\sim L/\delta$ results in a large Meissner magnetic moment $\sim (V_{eff}/4\pi)(L/\delta)H\theta$, whose projection parallel to **H** is a factor $\sim \theta^2 L/\delta$ relative to the signal of interest. Even for $\theta \sim 0.2°$ both contributions are of the same order, thus completely invalidating the interpretation of the data. Moreover, spurious perpendicular vortex penetration (which does not happen in real cavities) occurs at fields $\sim (\delta/\theta L)H_{c1}$, which can be as low as a few Oe.

In the ellipsoids the misalignment produces a transverse Meissner magnetic moment $VH\theta/[4\pi(1-N_b)]$, whose projection parallel to **H** is a factor $\sim \theta^2(1-N_a)/(1-N_b) \sim \theta^2$ relative to $m_{M,a}$. This is just a minor correction, thus the ellipsoid volume can be accurately obtained and the results will be reproducible even if the alignment changes slightly between measurements. Finally, the

misalignment does not produce transverse vortex penetration at very low fields because the demagnetizing factors for $H_{\perp}$ and $H_{||}$ are of the same order. The highest field (and hence the first vortex penetration) occurs at the two diametrically opposed points at the intersection of the equatorial lines for $H_{\perp}$ and $H_{||}$ and the magnitude of that field is $H_{eq}(\theta) = \{[H_{||}/(1-N_a)]^2+[H_{\perp}/(1-N_b)]^2\}^{1/2} \sim [H/(1-N_a)]\{1+\theta^2[(1-N_b)/(1-N_a)]^2/2\} \sim [H/(1-N_a)]\{1+\theta^2\}$, i.e., the misalignment only produces a small error of order θ^2 in the determination of H_{vp}.

MgB_2 films on Mo ellipsoids

Mo ellipsoids nominally identical to the Nb ellipsoids were also machined with computer-controlled lathes. We selected Mo (a non-superconducting metal) as a substrate for MgB_2 films based on its easy machinability and good performance. Since Mo is a very weak paramagnetic material with $\mu < 10^{-6}$, it serves as an ideal platform to study the superconducting MgB_2 shell itself.

Extended Data Fig. 1b shows $m(H)$ at several T for a MgB_2 film of thickness d_S = 100 nm deposited on top of a Mo ellipsoid, measured with $\mathbf{H}||a$ and following the protocol described in the main text. The first observation is that the full volume of the ellipsoid is screened, as demonstrated by the fact that the Meissner slope $m_{M,a}/H$ = -0.01043 emu/Oe is within 2% of the value for the Nb ellipsoid, the small difference being an indication of the reproducibility of the machining. Extended Data Fig. 1c shows $<B>$ vs. H, and the resulting $H_{vp}(T)$ (defined as the point where $<B>$ increases above the noise level) is shown in Extended Data Fig. 1d (both in terms of the applied H and H_{eq}). The $H_{vp}(T)$ curve extrapolates to zero at the transition temperature of the film (T_c ~ 38.1 K, as shown in the inset of Extended Data Fig. 1d). We coated additional Mo ellipsoids with MgB_2 films of thickness d_S = 200 and 300 nm. They show slightly higher T_c (38.6 and 38.7 K respectively), and in both cases we obtained Meissner slopes that correspond to a shielding of the

whole volume. As an example, Extended Data Fig. 1e shows <B> vs. H for all samples at selected temperatures, and all the $H_{vp}(T)$ data are shown in the Fig. 3a of the main text.

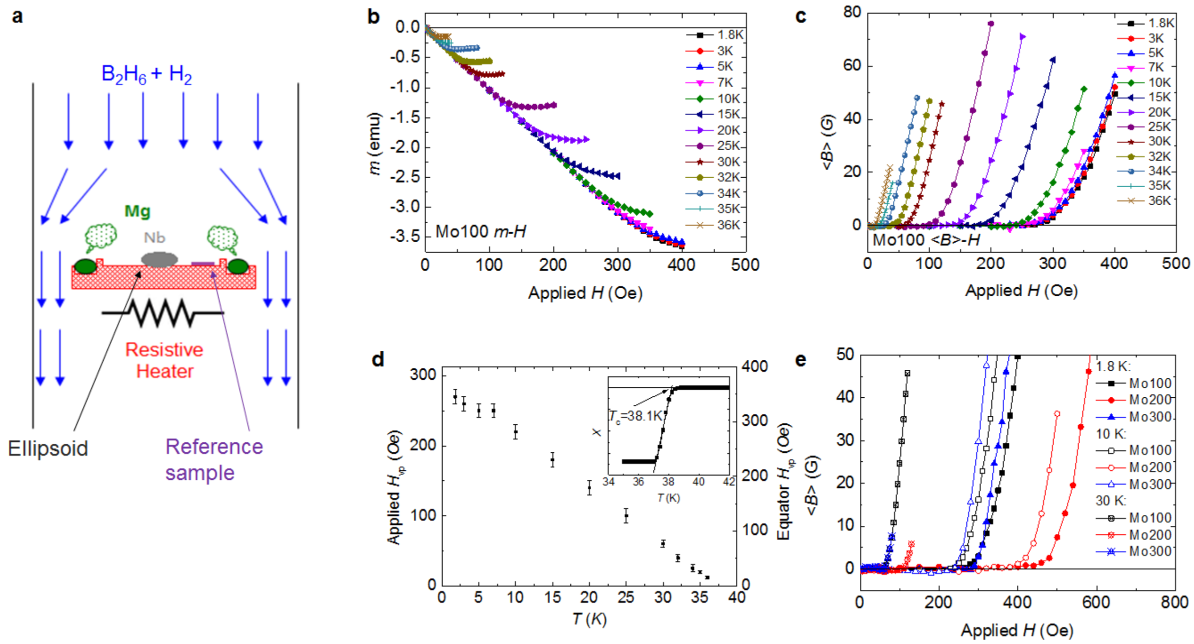

Extended Data Figure 1: Data for MgB_2 coated Mo ellipsoids. (a) Schematic of the HPCVD process. (b): ZFC m-H curves for Mo100 ellipsoid at different temperatures. (c): <B>-H curves for Mo100 ellipsoid at different temperatures. (d) Mo100's temperature dependence of H_{vp}, inset shows T_c = 38.1 K as measured via susceptibility measurement. (e) Average internal inductance <B> versus T curves of 3 ellipsoids at 3 different temperatures. Matching color curves correspond to the same ellipsoids while matching symbol shapes correlate to the same temperatures. (f): Summarized H_{vp}–T curves for Mo ellipsoids with different thicknesses of the MgB_2 coating.

We now discuss in more detail what takes place at $H_{vp}(T)$. An important difference between the bare Nb ellipsoid and the MgB_2-coated Mo ellipsoids is that in the Nb ellipsoid the penetration of vortices with increasing H is gradual, as the front of the critical state profile moves inward, thus making the transition from the linear $m(H)$ Meissner response to the critical state response

(approximately parabolic with the same initial slope) intrinsically difficult to determine [(see Fig. 1(b) and (c)]. In contrast, in the MgB_2-coated Mo ellipsoids the shielding is due only to the superconducting currents in the thin MgB_2 film. As soon as the field at the MgB_2-Mo interface becomes nonzero it spreads inside the whole volume, thus the deviation from the Meissner response is sharper [see Fig. Extended Data 1(b), (c) and (e)]. Thus, there is no risk of overestimating H_{vp} due to a gradual critical state evolution.

The next step is to compare H_{vp} in the MgB_2-coated Mo with the expected H_{c1} of the MgB_2 films. Films deposited on single crystal substrates in the same system under the same conditions have $\lambda(0) \sim 50$ nm and $\xi(0) \sim 7$ nm,[1,2] so in the bulk limit $H_{c1}(0) \sim (\Phi_0/4\pi\lambda^2)\ln(\kappa) \sim 1500$ Oe, much higher than H_{vp} for any of the samples in Extended Data Fig. 1f. On the other hand, if we estimate that $\lambda \sim 100$ nm or 140 nm as we did in the analysis of Fig. 3, for the bulk limit we obtain $H_{c1}(0) \sim 500$ Oe and 300 Oe respectively. Calculating the enhancement prediction with the magnetic field only on the outside[3], for $\lambda \sim 100$ nm the $H_{c1}(0)$ results are approximately 1600 Oe, 900 Oe and 700 Oe for $d_S = 100$ nm; 200 nm and 300 nm respectively, while for $\lambda \sim 140$ nm we obtain ~ 1200 Oe; 750 Oe and 540 Oe. Comparing these values with the experimental data in the Extended Data Fig. 1f, we conclude that in all cases H_{vp} at the equator is lower than the prediction for the enhanced H_{c1}. The interpretation is that the field penetration at H_{vp} indicates that the surface barrier vanishes, so vortices start to slide through the MgB_2 into the Mo. It is consistent with this picture that H_{vp} at the equator is dramatically lower than the enhanced H_{c1} in the $d_S = 100$ nm film (because the ΔH that it can sustain is the smallest) and less so in the thicker films.

Below H_{vp}, the shielding current flowing in the film reduces the field from the value at the external surface to exactly zero at the MgB_2/Mo interface. For the following discussion we will consider a

simpler geometry of an infinite flat film parallel to the *y*-*z* plane occupying the space from $x = 0$ to $x = d_S$, with finite $\mathbf{H} \| z$ for $x \geq d_S$ and $H = 0$ for $x \leq 0$ (see Extended Data Fig. 2a). Bearing in mind that d_S is much smaller than the axes of the ellipsoid this is a reasonable approximation for the field and current density distribution inside the film at the equator, so

$$h(x) = \frac{H}{1-N_a}\frac{\sinh\left(x/\lambda\right)}{\sinh\left(d_S/\lambda\right)} \qquad (1)$$

$$J(x) = \frac{c}{4\pi}\frac{H}{(1-N_a)\lambda}\frac{\cosh\left(x/\lambda\right)}{\sinh\left(d_S/\lambda\right)} \qquad (2)$$

The maximum density always occurs at the external surface, $J_{max} = J(x = d_S) = \frac{c}{4\pi}\frac{H}{(1-N_a)\lambda}\coth\left(d_S/\lambda\right)$, but the current becomes more uniform as δ/λ decreases. The penetration occurs when J_{max} is large enough to eliminate the surface barrier, i.e., to exert an inward force on the vortex that overcomes the attraction between the vortex and the anti-vortex image, and given ideal surface conditions this should occur approximately when $J_{max} \sim J_d$. For comparison, if we consider $\lambda(0) \sim 100$ nm, at $T \sim 1.8$ K we obtain $J_{max} \sim 37$; 45 and 28 MA/cm^2 for $d_S = 100$; 200 and 300 nm respectively. These are very large values, more than 20% of J_d.

The fact that H_{vp} in the MgB_2 coated Mo ellipsoids is determined by J_{max} rather than H_{c1} implies that H_{vp} is the limit for the *difference* in field that can be sustained between both surfaces of the film, not for the *absolute value* of the field. This is fully consistent with our main experimental finding, namely that by adding an MgB_2 coating we have been able to increase H_{vp} by about 500 Oe on top of the already large penetration field of Nb. In order to estimate how high is the H_{vp} that could potentially be obtained with this method, there is one more test that we can perform on the MgB_2 coated Mo samples.

Extended Data Figure 2b shows the schematic of the measurement. First we apply a magnetic field at a temperature above T_c. We call this the internal field, H_{int}, because it penetrates into the Mo, and is homogeneous to the extent that the susceptibility of the Mo and the MgB_2 in the normal state are negligible. Next we field cool (FC) in the presence of H_{int} to a measurement temperature T. As the MgB_2 becomes superconducting currents will develop to try to expel the field from inside the film, but not from inside the Mo, where the field will remain essentially uniform and equal to H_{int}. These currents will have opposite direction in the inside and outside surfaces of the MgB_2 film and will produce a very small net magnetic moment (at the most a Meissner state inside the film). Extended Data Fig. 2c shows an example where H_{int} = 1000 Oe and T = 5 K for the 200 nm film. The initial point measured after FC indeed has $m = 0$ within the resolution (several orders of magnitude lower than the Meissner signal of the Nb ellipsoid at that field). Now we increase H and measure $m(H)$. Although the field range is well above the H_{vp} shown in Extended Data Fig. 1f, we observe a linear dependence with the same slope as in the Meissner response after ZFC. This indicates that screening currents develop in the MgB_2 that shield the internal Mo volume from the external field variation. As the field difference between the outside and the inside $\Delta H = H\text{-}H_{int}$ increases, eventually the surface barrier disappears and the field starts to penetrate; we call this difference ΔH_{vp}. Extended Data Fig. 2d shows $(m\text{-}m_M)$ vs. ΔH after FC at several H_{int}, for the same sample and T than Extended Data Fig. 2c. Initially $m\text{-}m_M = 0$ ("Meissner-like" response) and deviates at a H_{int}-dependent ΔH_{vp}, as summarized in Fig. 3b of the main text. In particular, the points for $H_{int} = 0$ in Extended Data Fig. 2e correspond to the ZFC condition and coincide with the data at the same temperature in Extended Data Fig. 1f.

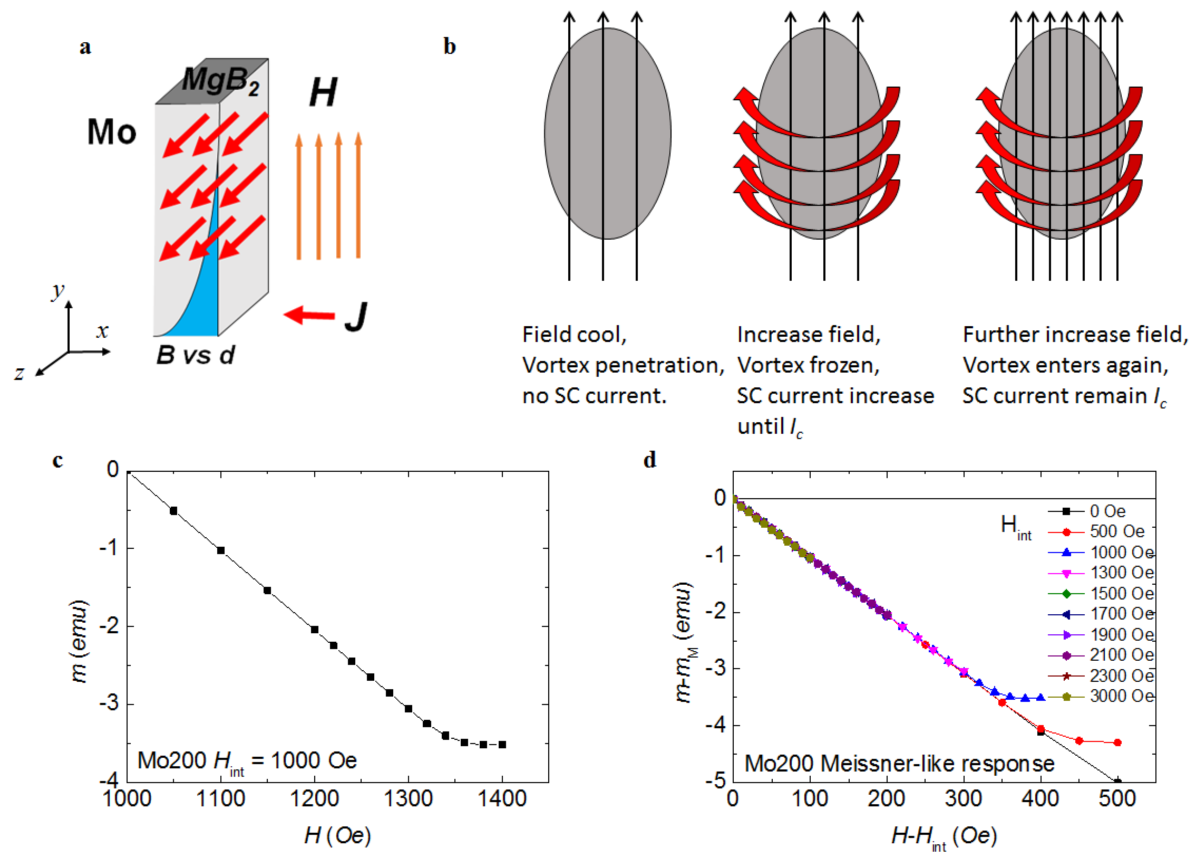

Extended Data Figure 2: (a) Schematic of the FC Meissner-like measurement. (b) Field distribution and current distribution in the MgB_2 shell under increasing field. (c) *m*-*H* curve for Mo200 ellipsoid after 1000 Oe field-cool. (d): Δm-ΔH curves for Mo200 ellipsoid at 1.8 K after field-cooling with different H_{int}. (e) ΔH_{vp} versus temperature curve for Mo200 at 1.8 K and 5 K.

m-H measurements of MgB_2 films coated Nb ellipsoids

Extended Data Fig. 3a shows $m(H)$ at several T for a MgB_2 film of thickness δ = 200 nm deposited on top of a Nb ellipsoid. The Meissner slope (-0.00988 emu/Oe) indicates full volume screening, and H_{vp} below the T_c of Nb is higher than in the bare Nb ellipsoid. To observe this enhancement more clearly, Fig. 3b shows the comparison of <*B*> vs. *H* for the MgB_2-coated Nb and bare Nb ellipsoids at selected temperatures. At T = 9 K, the H_{vp} for bare Nb ellipsoid is less than 50 Oe and increases to 200 Oe on Nb100 and 400 Oe on Nb200 ellipsoids. However, at T = 1.8 K, the H_{vp}

comparison among the 3 ellipsoids is difficult. Anomalous $\langle B \rangle$ fluctuation happens at 2000 Oe and blurs the turning points on the $\langle B \rangle$-H curves that indicate vortex penetration. As discussed in the main text, the 2 G noise level at 2000 Oe makes the m-H method not suitable for measuring the high H_{vp} of Nb and coated Nb ellipsoids.

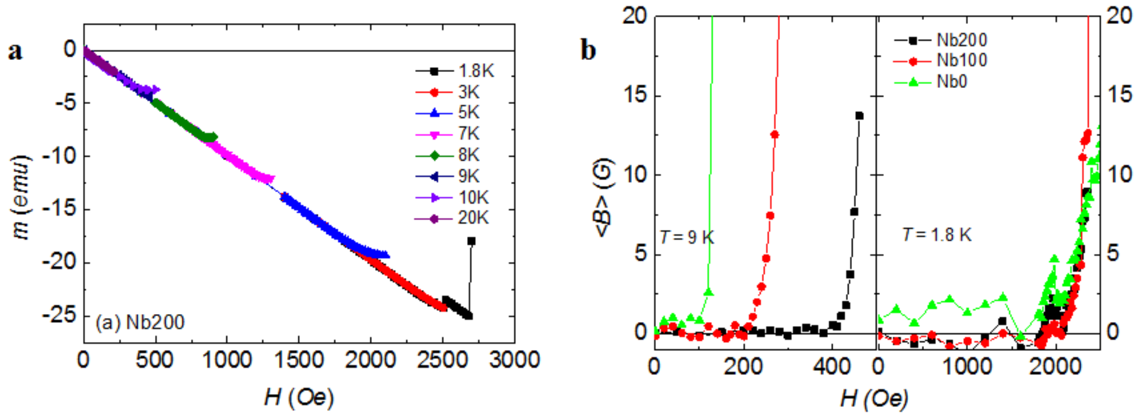

Extended Data Figure 3: (a): m-H curve for Nb200 ellipsoid at different temperatures. (b): $\langle B \rangle$-H curve comparison between Nb ellipsoids with different MgB_2 coating thicknesses. Left column shows curves at T = 9 K, while right column shows curves at T = 1.8 K.

Theoretical analysis of the MgB_2/Nb structure

Assuming the surface of the MgB_2-claded Nb ellipsoid is ideal, we performed a conservative estimation about the H_{sh} of MgB_2 with the method described in reference [16]. The lowest H_{sh} of MgB_2 which can produce the H_{vp} measured in this work is ~3000 Oe. The relationships between $H_{vp}(T=0)$ and d_S calculated for the MgB_2/Nb structure without insulating layer (d_I=0) are plotted in Extended Data Figure 4 for λ_{MgB2} = 100 nm (red) and 140 nm (blue). The solid stars in Fig. 3a indicate the H_{vp} for Nb100 and Nb200 at the lowest measured temperatures (2.3K and 2.8K respectively) and the open stars are the estimated $H_{vp}(T=0)$ obtained by linearly extrapolating the H_{vp} – T curves in Fig. 2d to 0 K. Because the surface of the MgB_2 coated Nb ellipsoid is far from

ideal, and thin MgB_2 films on Nb always have reduced T_c and H_c compairing with bulk materials,[13] a higher $H_{sh}(0)$ for MgB_2 should be expected.

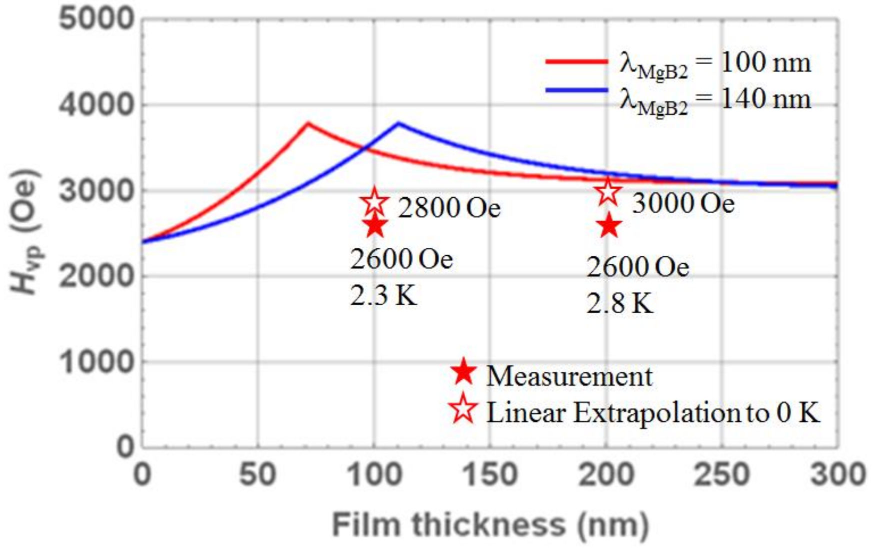

Extended Data Figure 4: H_{vp} as a function of d_S. Filled stars show our measurement results and the corresponding temperatures. The open stars show the linear extrapolation of H_{vp} to 0 K.

References cited:

1 Cunnane, D. *et al.* Penetration depth of MgB2 measured using Josephson junctions and SQUIDs. *Appl Phys Lett* **102**, 072603, doi:10.1063/1.4795244 (2013).

2 Xi, X. X. MgB2 thin films. *Supercond Sci Tech* **22**, 043001, doi:10.1088/0953-2048/22/4/043001 (2009).

3 Hein, M. *High-Temperature-Superconductor Thin Films at Microwave Frequencies*. (Springer Berlin Heidelberg, 1999).
